# Sprayable Diacetylene-Containing Amphiphile Coatings for Visual Detection of Gas-Phase Hydrogen Peroxide

**DOI:** 10.3390/chemosensors12050071

**Published:** 2024-05-01

**Authors:** Priyanka Shiveshwarkar, Justyn Jaworski

**Affiliations:** Department of Bioengineering, University of Texas at Arlington, Arlington, TX 76010, USA;

**Keywords:** sprayable sensor, hydrogen peroxide vapor, polydiacetylene, iron chloride

## Abstract

Colorimetric chemical sensing of target gases, such as hydrogen peroxide vapors, is an evolving area of research that implements responsive materials that undergo molecule-specific interaction, resulting in a visible color change. Due to the intuitive nature of an observable color change, such sensing systems are particularly desirable as they can be widely deployed at low cost and without the need for complex analytical instrumentation. In this work, we describe our development of a new spray-on sensing material that can provide a colorimetric response to the presence of a gas-phase target, specifically hydrogen peroxide vapor. By providing a cumulative response over time, we identified that part per million concentrations of hydrogen peroxide vapor can be detected. Specifically, we make use of iron chloride-containing formulations to enable the catalysis of hydrogen peroxide to hydroxyl radicals that serve to initiate polymerization of the diacetylene-containing amphiphile, resulting in a white to blue color transition. Due to the irreversible nature of the color change mechanism, the cumulative exposure to hydrogen peroxide over time is demonstrated, enabling longitudinal assessment of target exposure with the same coatings. The versatility of this approach in generating a colorimetric response to hydrogen peroxide vapor may find practical applications for environmental monitoring, diagnostics, or even industrial safety.

## 1. Introduction

The detection of hydrogen peroxide (H_2_O_2_) has implications for industry (i.e., process control, leak detection, and sterilization), environmental safety (i.e., assessing occupational exposure or contamination of wastewater), and human health (i.e., high H_2_O_2_ levels in exhaled breath are associated with certain respiratory diseases) [1–4]. Research on methods for the detection of hydrogen peroxide has, thus, been very active for both liquid-phase sensing of H_2_O_2_ as well as gas-phase sensing of H_2_O_2_ vapor. Comparatively, the detection of H_2_O_2_ within liquid samples has seen markedly higher attention and has seen particular success with electrochemical sensing mechanisms involving reduction–oxidation processes stemming from H_2_O_2_ dissociation [5]. Such electron transfer processes provide an appropriate source for electrochemical-based sensing of H_2_O_2_, and similarly small-molecule fluorophores undergoing electron transfer reactions with H_2_O_2_ (or decomposition products thereof) can facilitate sensitive detection [6–8]. Small-molecule-based fluorescent probes for liquid-phase detection of H_2_O_2_ by mechanisms including Baeyer–Villiger oxidation rearrangements, oxidation of phenols and boronate-based probes, and metal-mediated redox reactions, among others, have been extensively reviewed [9]. Systems for liquid-phase detection of H_2_O_2_ have also been developed that provide a colorimetric (visible color change) response, wherein such systems employ polydiacetylene-based vesicles [10–12].

In contrast to liquid-phase sensing, the detection of H_2_O_2_ vapor possess additional challenges due to the typically lower concentrations. To circumvent such issues, groups have implemented preconcentration methods and even condensation of the gas to the liquid phase to allow spectroscopic or electrochemical analysis of H_2_O_2_ content within the condensates, which is particularly common for exhaled-breath analysis [13]. True gas-phase detection of H_2_O_2_ has, however, seen relatively fewer reports, but among those the most common platforms are based on metal oxide semiconductor gas sensors [14–17]. One such system for direct detection of H_2_O_2_ vapor concentrations utilizes CuO, which exhibits a reversible change in the resistance behavior to H_2_O_2_ at high concentration ranges of 2–7% *υ*/*υ* (which are concentrations relevant to packaging sterilization processes) [16]. In another example, cobalt-doped tin dioxide films demonstrated a working range of approximately 500 ppm to 1700 ppm of H_2_O_2_ [18]. MnO_2_ has also been used as the active element for the decomposition of hydrogen peroxide on a calorimetric-based sensing platform, showing detection in the range of approximately 2–7% *υ*/*υ* H_2_O_2_ [14]. While many of these systems operate at elevated temperatures, detection of hydrogen peroxide gas at room temperature was demonstrated using a sensor design utilizing Pt nanoparticles decorated on a single-walled carbon nanotube network film, which yielded resistance changes across the range of 2 to 60 ppm H_2_O_2_ [15]. Chronoamperometry-based detection of H_2_O_2_ has recently demonstrated even lower detection limits, in the range of 50 to 800 ppb, using a gold-nanoparticle-modified nafion membrane with room temperature ionic liquid for high-surface-area gas absorption [19]. Another recent electrochemical study for gas-phase detection of H_2_O_2_ utilized a screen-printed electrode system, comprising a polyacrylic acid-based sensing membrane, which exhibited readable voltametric signals to trace levels of hydrogen peroxide gas, approximately 7 ppb (10 μg/m^3^) after a 40 min accumulation time [20]. Enzyme-embedded electrochemical systems for capacitive monitoring of H_2_O_2_ vapor have also been developed and have yielded reliable detection across the range of 116 to 630 ppm [21].

As an alternative to the aforementioned H_2_O_2_ gas detection systems, in this work we are examining a colorimetric spray-on sensor that does not require complex instrumentation or analysis. A notable example of an existing colorimetric approach to hydrogen peroxide vapor detection that does not rely on electrical readout makes use of ammonium titanyl oxalate drop-cast onto paper to provide a color change response to H_2_O_2_ gas [17]. This impressively simple and easy-to-read approach facilitates a distinct visual transformation to a yellow color upon exposure to the vapor from 35 wt % H_2_O_2_, and when used with color analysis equipment, H_2_O_2_ vapor as low as 0.4 ppm could be detected [17]. In expanding this paradigm for manufacturing a widely deployable and easily interpreted colorimetric sensing system, we have developed a sprayable sensor that enables coating onto surfaces for visual assessment of the presence of hydrogen peroxide gas and can enable determination of the extent of cumulative exposure. By enabling surfaces with complex shapes to be coated with this sensing platform, the detection approach provided herein could afford wearable sensors for detecting hydrogen peroxide exposure or if implemented onto static surfaces could be used for broad spatial mapping of variations in H_2_O_2_ gas concentrations within regions of interest.

**Figure 1.**
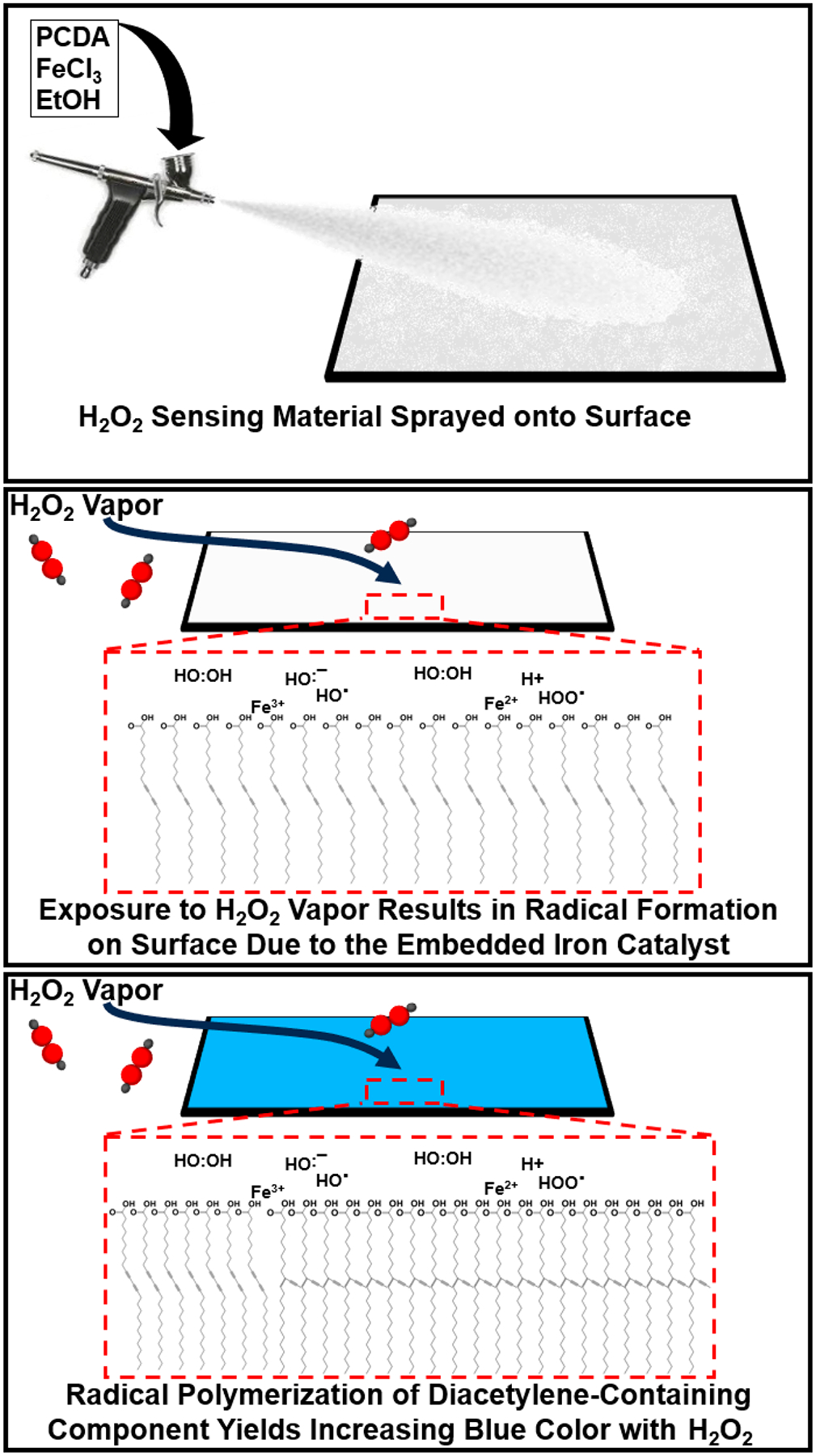
Generalized overview of spray-on sensor application (**top**); exposure to H_2_O_2_ resulting in radical formation by reacting with iron (**middle**); and radical-initiated polymerization of diacetylene-containing monomers resulting in surface appearing blue (**bottom**).

As depicted schematically in Figure 1, our spray-on sensing system relies on a formulation possessing diacetylene-containing amphiphiles to enable the visual transformation from colorless to blue upon polymerization to polydiacetylene. The inclusion of an iron catalyst enables decomposition of hydrogen peroxide vapor to hydroxyl radicals that initiate the polymerization process, resulting in the associated increase in the observed blue color with cumulative hydrogen peroxide exposure. While several recent works looking at the detection of hydrogen peroxide with polydiacetylene have demonstrated liquid-phase detection of hydrogen peroxide using polydiacetylene vesicles [10–12,22], here we provide the first report of colorimetric detection of gas-phase hydrogen peroxide using spray-on diacetylene amphiphile formulations. We have previously demonstrated that diacetylene-containing amphiphile derivatives could provide a platform for generating sprayable coatings on various surfaces to convert those surfaces into color-changing sensors for different chemical and physical stimuli [23,24]. In contrast to this prior work, here.

We demonstrate new spray coating formulations that can provide a visual response to low-ppm levels of hydrogen peroxide. This characteristic of the spray-on sensor enables the potential for not only assessing acute hydrogen peroxide vapor levels but also for use for periods of extended duration, allowing long-term cumulative hydrogen peroxide exposure to be determined. Because the amphiphile and iron content of the spray-on formulations can be customized to provide a distinct color gradient profile specific to the cumulative amount of hydrogen peroxide gas exposure, we were able to generate spray coating variations with different degrees of sensitivity to hydrogen peroxide gas. For example, cumulative detection of continuous exposure to 94 ppm of hydrogen peroxide vapor was still demonstrating appreciable color changes even after 4 days of observation. The lower limits of detection identified in our approach were in the range of 0.2 to 3 ppm after 2 h of exposure. These part-per-million (ppm) levels of hydrogen peroxide detection in the gas phase are comparable with those reported by the electrochemical and colorimetric systems described above. A major benefit of this approach is the ease of applying the sensor onto a variety of surfaces, including those with complex geometries, and over areas as large as desired to allow data of hydrogen peroxide vapor exposure to be collected, imaged, or mapped spatially without the need for expensive or complex sensor architectures.

## 2. Materials and Methods

### 2.1. Preparing PCDA-Containing Spray Formulation

Initially, 10,12-pentacosadiynoic acid (PCDA) amphiphile monomers were separated from polymerized polydiacetylene by dissolving the purchased PCDA (Sigma Aldrich, St. Louis, MO, USA) in methylene chloride and passing the mixture twice through cotton compressed in a Pasteur pipette to serve as a filter. The purified solution of PCDA monomers was transferred to a round-bottom flask wherein the methylene chloride was evaporated using a rotary evaporator at 50 °C, 700 torr. The white colored powder of monomeric PCDA was then stored at 4 °C in a dark, amber bottle to prevent polymerization. For generation of different spray-on sensor formulations with varying amounts of PCDA amphiphile and iron content, we freshly prepared a stock solution of 30 mg/mL of PCDA in 100% ethanol and also a 1 M iron(III) chloride stock solution in water. The stock PCDA amphiphile solution in ethanol was sonicated for 1 min. Serial dilutions of the iron(III) chloride solution were prepared in water to provide the original 1 M stock as well as 100 mM, 10 mM, and 0.01 mM iron(III) chloride. Ethanol served as the carrier solvent for spray application of the sensor formulations; therefore, dilutions of the PCDA amphiphile were prepared in 100% ethanol to provide concentrations including the original 30 mg/mL PCDA as well as 20 mg/mL, 5 mg/mL, 2.5 mg/mL, and 1 mg/mL. The formulations were prepared by mixing 1 mL of selected PCDA dilutions with 10 μL of selected iron(III) chloride dilutions followed by sonication of the mixture for 1 min and, prior to airbrushing, 30 s of mixing using a vortexer to ensure proper mixing of the sample formulations.

### 2.2. Spray Processing of Formulations

The freshly prepared formulations of PCDA and iron(III) chloride-containing solutions prepared in ethanol were loaded into a reservoir attached to an NEO BCN Siphon Feed Dual Action Airbrush. In this system, air was delivered by an Iwata Ninja Jet Airbrush Compressor (ANEST Iwata-Medea Inc., Portland, OR, USA) in order to allow the formulations to be uniformly spray-coated onto a surface. Here, we utilized grade 1 Whatman filter paper cut into 5 cm × 2 cm pieces on which 1 mL of formulation was airbrushed to provide a homogeneous coating on the surface of the filter paper. During spray coating of the paper substrates, the solution was applied in two coating steps, with 500 μL applied each time to ensure uniform and homogeneous coating of the substrates. The airbrush nozzle was directed at a 90° angle (orthogonal with respect to the substrate) for spraying with a side-to-side motion at a distance of 12 cm from the substrate when applying the amphiphile-containing solution onto the filter paper substrate. The spray-coated surface was allowed to air-dry for several minutes until the ethanol had completely evaporated. These spray-coated sensors were then either used once dry or stored for later use in an air-tight container. The container was place in a dark location to ensure the coatings did not polymerize due to UV exposure as UV light is capable of photopolymerizing diacetylene-containing amphiphiles.

### 2.3. Examining the Response of Spray-Coated Paper to Hydrogen Peroxide Vapor

Each formulation that was spray-coated onto filter paper was examined in terms of its color response to exposure to different concentrations of hydrogen peroxide vapor. The spray-coated paper was cut into smaller pieces (approximately 1 cm × 1 cm), and the initial colors of these sensors were recorded by taking a digital photograph immediately prior to exposure to the target hydrogen peroxide vapor. The sensors were placed over a small vessel containing 100 μL of hydrogen peroxide of a distinct dilution (dilutions in pure water provided H_2_O_2_ at 30%, 20%, 10%, 7.5%, 5%, 2.5%, 1%, 0.1%, 0.01%, 0.001%, and 0.0001%, which were converted to distinct saturated equilibrium headspace H_2_O_2_ vapor concentrations of 94 ppm, 64 ppm, 31 ppm, 23 ppm, 15 ppm, 7 ppm, 3 ppm, 0.2 ppm, 0.01 ppm, 0.001 ppm, and 0.00008 ppm, respectively, as based on reports of the vapor–liquid equilibrium in aqueous mixtures of hydrogen peroxide) [17,25,26]. Controlled experiments were carried out to measure the time-dependent visual color change of the sensors for each H_2_O_2_ vapor concentration. Experiments were conducted in a darkroom to prevent the possibility of UV photopolymerization. Photographs of the sensors were taken periodically to record the color change of the sensors in response to the cumulative hydrogen peroxide vapor exposure in the headspace. For all experiments, the digital photographs were taken using a smartphone with a 12-megapixel camera mounted at a constant distance and with consistent lighting. The images were analyzed using ImageJ and the analyze function in the plugins menu was used to extract the corresponding red–green–blue (RGB) values of the sensor, which were recorded and converted to CIELab colorspace values a*, b*, and 100-L for determining the extent of color change. The color change response of visual sensors has been previously represented in CIELab colorspace and is considered more representative than visual perception of color [17]. Compared to blue-channel image data alone, the CIELab colorspace encompasses more complete data for color changes that are more complex, for example, in this case, in which the color change appears to become both bluer and darker at the same time. Thus, the color change of the sensor was quantified by the change in the Euclidian distance of the CIELab colorspace for the a*, b*, and 100-L values.

## 3. Results

### 3.1. Assessment of Color Response as a Function of Cumulative Exposure to Hydrogen Peroxide Vapor over Time

**Figure 2.**
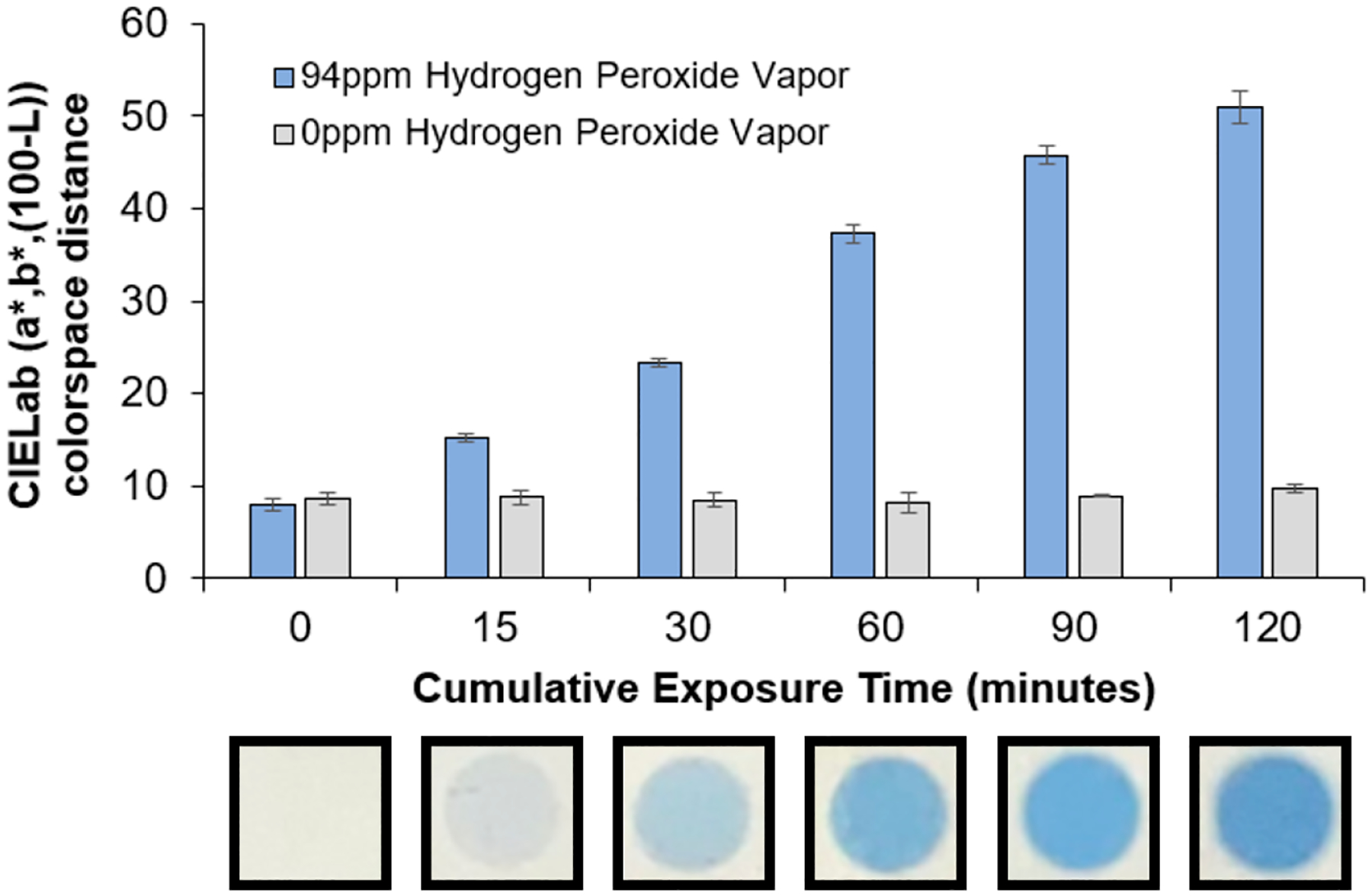
Demonstration of the color response of the spray-on formulation (20 mg/mL PCDA and 10 mM iron(III) chloride) to serve in the detection of the gas-phase hydrogen peroxide vapor as a function of time with the resulting color change following an increase in the CIELab colorspace distance with increasing cumulative exposure.

Here, we examined the first gas-phase detection of hydrogen peroxide vapor using a spray-on colorimetric sensor. The response provided by the spray coating appears as a visual blue color due to polymerization of diacetylene monomers to form conjugated polymers with distinct absorbance of visible light. The following investigation provides an assessment of spray-on formulations containing diacetylene monomers and iron for the conversion of hydrogen peroxide vapors to hydroxyl radicals, wherein this radical formation serves to initiate the polymerization of the diacetylene monomers. Our initial experiments examined the time dependence in the color change for a formulation containing a final concentration of 20 mg/mL of PCDA and 10 mM iron(III) chloride. As seen in Figure 2, the blue color appeared darker with an increase in the cumulative exposure time to the target gas of hydrogen peroxide vapor at 94 ppm. The first sample assessment at 15 min begins to provide a visually discernable blue color. This color change for the spray-on formulation was quantified using the CIELab colorspace distance and compared to control samples not exposed to H_2_O_2_, which showed no response over the course of the 2 h experiment. This result revealed that the spray-on sensor could facilitate a unique degree of blue color depending on the extent of hydrogen peroxide vapor exposure.

### 3.2. Impact of Formulations’ Iron(III) Chloride Content on Sensitivity of Color Response to Hydrogen Peroxide Vapor

**Figure 3.**
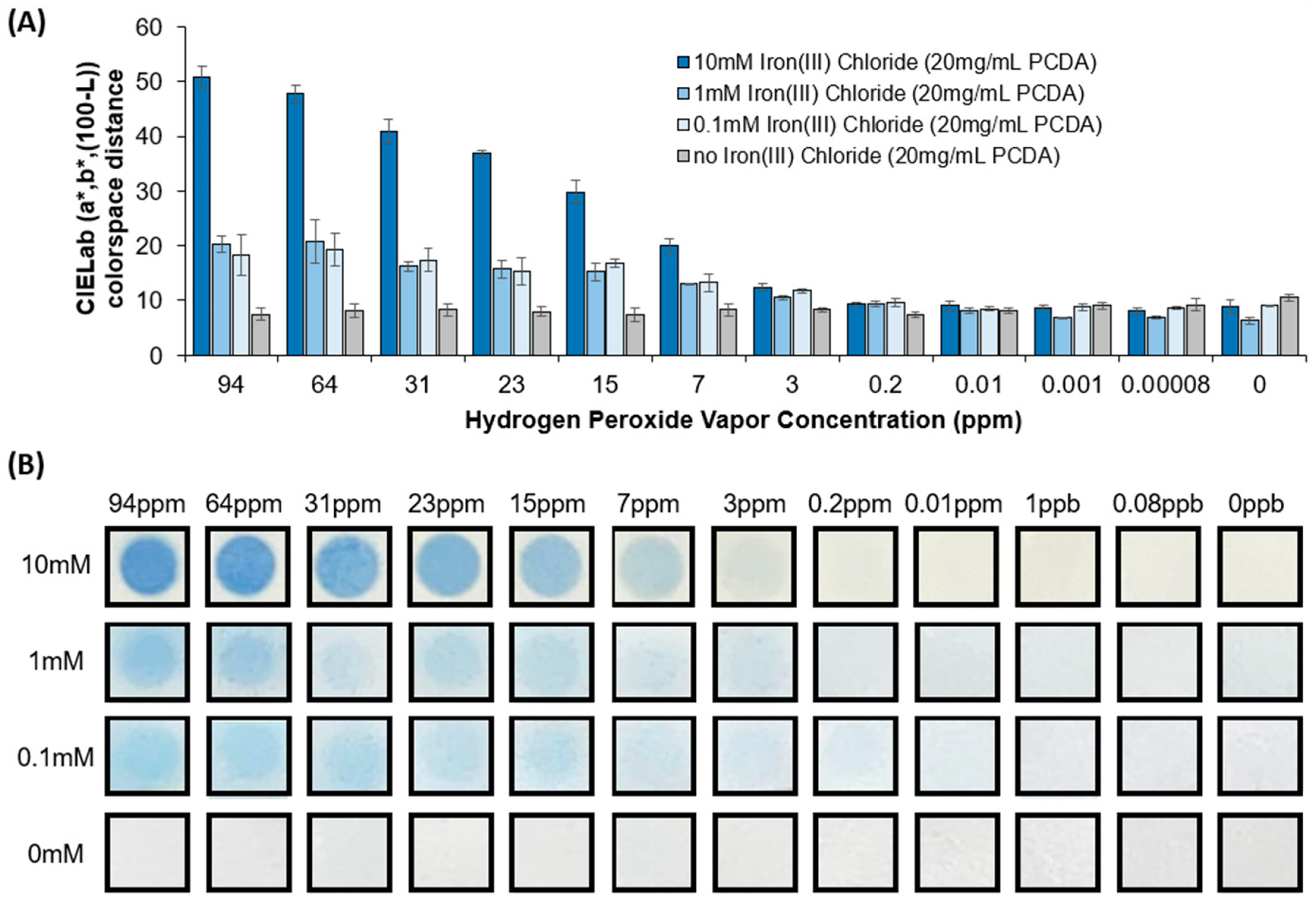
(**A**) CIELab colorspace distance of the blue color change observed in the spray-coated sensors, and (**B**) images of the spray-coated sensors (using formulations of 20 mg/mL PCDA with distinct iron(III) chloride contents of 0, 0.1, 1, or 10 mM) after exposure to various concentrations of hydrogen peroxide vapor for 2 h.

In order to assess the two components of our formulations, we first modulated the iron(III) chloride concentration using a constant 20 mg/mL PCDA concentration. As seen from our investigation in Figure 3, after 2 h of exposure to varying concentrations of hydrogen peroxide vapor, formulations with no iron(III) chloride did not provide a color response to hydrogen peroxide vapor at any of the concentrations tested. In contrast, utilization of a concentration as low as 0.1 mM iron(III) chloride in the formulation was found to be sufficient to provide an appreciable increase in the blue color when increasing the concentration of the target hydrogen peroxide vapor. The color response appeared to be significantly greater when implementing the highest concentration of 10 mM iron(III) chloride. It is also clear from the figure that the higher-iron-containing formulations provided a more distinct contrast in the exposed regions, wherein the sensing surface exposed to the hydrogen peroxide vapor headspace is at the circular opening of the vial. We can see that the hydrogen peroxide vapor resulted in a blue color formation which was related to the concentration of the iron(III) chloride component in the formulation. Despite these differences, it still appears by visual inspection, and also from the CIELab colorspace values, that the 3 ppm, and to some extent 0.2 ppm, have a blue color above that of the control samples, suggesting an observable limit of detection in the range of 0.2 to 3 ppm of hydrogen peroxide vapor. From Figure 3B, we can also see comparatively that the coatings with 1 mM iron and 0.1 mM iron content exhibited a more diffuse, scattered blue response as compared to the higher-content 10 mM iron formulations, which provided a sharp blue response at the hydrogen peroxide exposure boundary. We suspect this diffuse response to be a physical manifestation of the sensor response depending on radical polymerization, in that a higher iron content would in turn provide a higher density of sites for termination of radical polymerization through reduction of iron, while a lower iron content would provide lower rates of deactivation that may allow the observed diffuse appearance of a blue color response beyond the immediate boundary of exposure.

### 3.3. Impact of Formulations’ PCDA Concentration on Sensitivity of Color Response to Hydrogen Peroxide Vapor

**Figure 4.**
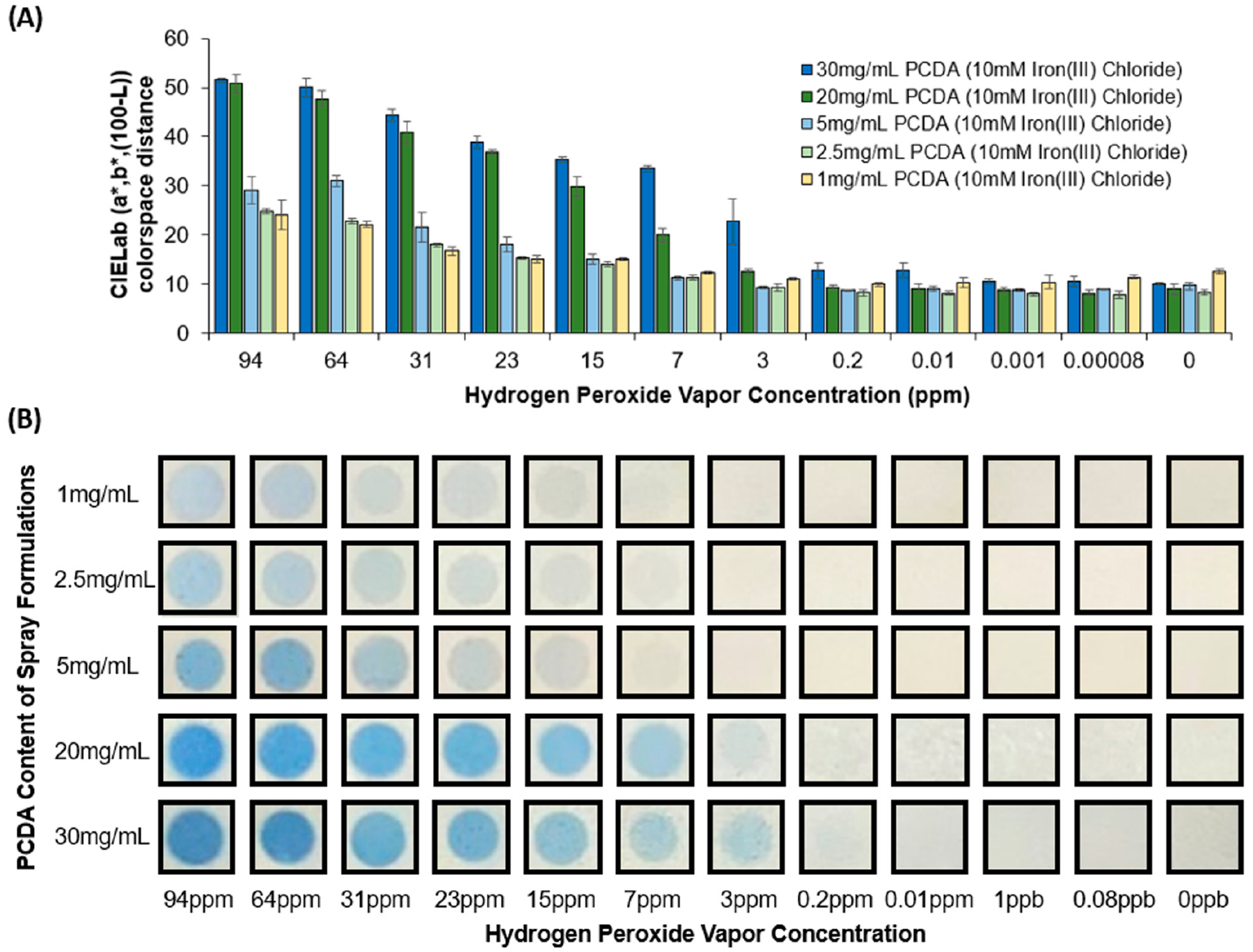
(**A**) CIELab colorspace distance of the blue color change observed in the spray-coated sensors and (**B**) images of the spray-coated sensors (using formulations of 10 mM iron(III) chloride and different PCDA contents of 1, 2.5, 5, 20, or 30 mg/mL) after exposure to various concentrations of hydrogen peroxide vapor for 2 h.

In our next set of experiments, we modified the concentration of the diacetylene-containing amphiphiles in the formulations in the range of 1 mg/mL to 30 mg/mL of PCDA. As shown in Figure 4, exposing the different formulations of the spray-on sensors to the same amount of hydrogen peroxide vapor resulted in a distinct blue color formation which was dependent on the concentration of the diacetylene-containing component in the formulation. From these results, it can be seen that higher PCDA concentrations in the formulation provided superior color response, even at low hydrogen peroxide concentrations. The hydrogen peroxide vapor detection limit for the higher-PCDA-content formulations (20 and 30 mg/mL) appeared to be 3 ppm and 0.2 ppm, respectively. In contrast, when using 1 to 5 mg/mL of PCDA in the formulation, the detection limit was between 7 and 15 ppm. In addition, the extent of the color response across the H_2_O_2_ vapor concentration range tested provided a substantially larger difference in color when using the higher-PCDA-content formulations. From this, we can observe that the concentration of the amphiphile and the iron content (as shown in the prior figure) can be tailored to provide a distinct color response profile specific to the cumulative amount of H_2_O_2_ exposure.

### 3.4. Assessing Long-Term Cumulative Exposure to Hydrogen Peroxide Vapor for Different Formulations

**Figure 5.**
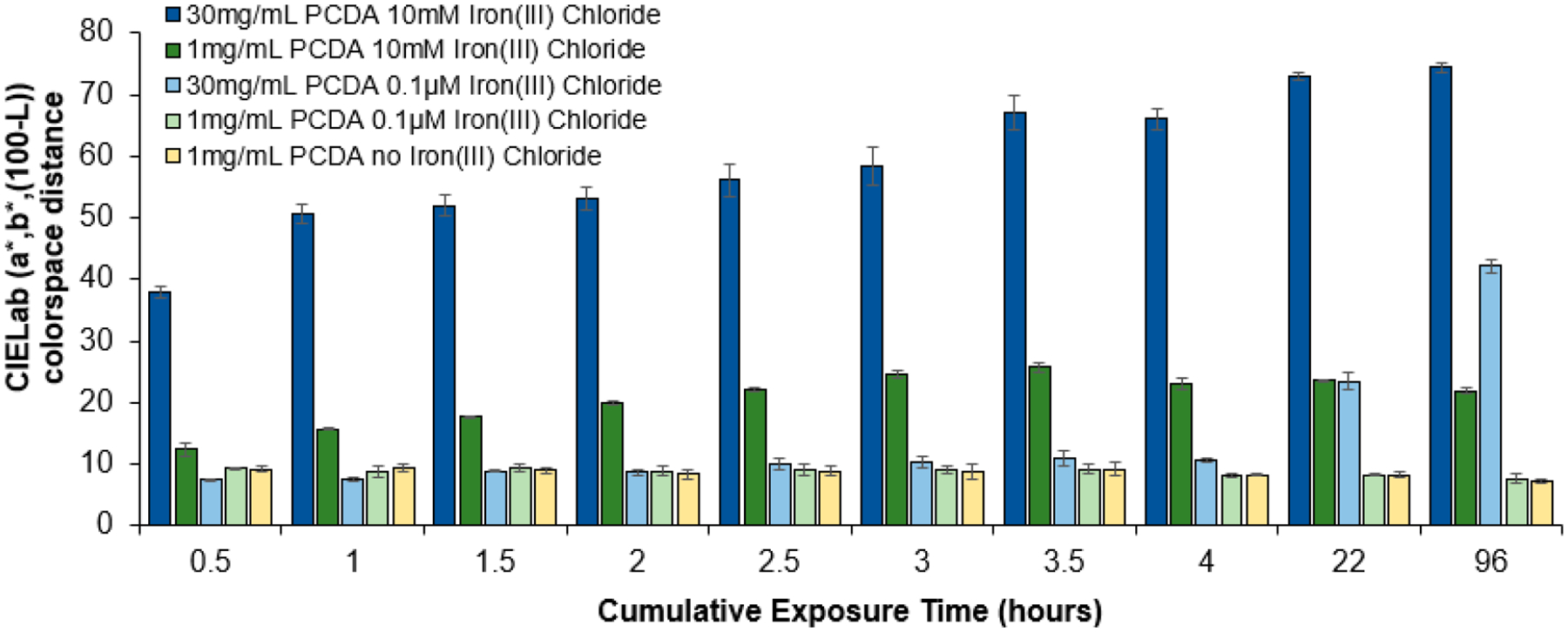
CIELab colorspace distance showing the color response of the sensors over an extended duration of 4 days (96 h) of exposure to 94 ppm of hydrogen peroxide vapor. Sensor formulations used were at the extremes of PCDA and iron(III) chloride contents, with high values of 30 mg/mL PCDA and 10 mM iron(III) chloride, while low values were 1 mg/mL PCDA and 0.1 μM iron(III) chloride. A non-responsive control formulation with no iron(III) chloride is provided for comparison.

In our last set of experiments, we examined the extreme formulation cases of high PCDA content (30 mg/mL) and low PCDA content (1 mg/mL) with a high iron(III) chloride content of 10 mM and a very low iron(III) chloride content of 0.1 μM in order to assess aspects of longitudinal assessment over longer timescales of cumulative exposure to 94 ppm hydrogen peroxide vapor. Specifically, these experiments were conducted to inform the upper limit point of saturation of the sensor response to cumulative exposure (using the high PCDA and iron(III) chloride formulations) and to assess if even slower color responses could be used to allow for extended studies for several hours or even days. As seen in Figure 5, we can clearly see that the 30 mg/mL PCDA formulation with 10 mM iron(III) chloride continued to increase up to 3.5 to 4 h, after which it appeared to saturate in its response. Lowering the amount of iron(III) chloride catalyst to 0.1 μM and using the same 30 mg/mL PCDA, however, allowed a slower color response that continued to increase with cumulative exposure even up to the 96 h (4 day) timepoint of this study. In looking at the 1 mg/mL PCDA formulation with 10 mM iron(III) chloride, we also see that saturation was reached quickly, within 3.5 to 4 h of exposure to the 94 ppm hydrogen peroxide. In this case, the ultimate color response at the saturation point for the 1 mg/mL formulation was smaller than that of the 30 mg/mL formulation, which gave a darker color at saturation. The 1 mg/mL PCDA formulation with 0.1 μM iron(III) chloride provided no color response over the course of the study and was the same as that of the control with no iron chloride. From the figure it is clearly observed that an increase in response over time based on the cumulative exposure seems to saturate quickly within 3.5 to 4 h when high concentrations of iron(III) chloride are used; however, when using a lower concentration of 0.1 μM of iron(III) chloride the response is slower, allowing for longer-term studies of cumulative exposure to be conducted. Because the measurements were taken at 30 min time points up to the 4 h mark, we could see the gradual increase in signal until reaching the maximal response near the 3.5 to 4 h mark for the high-iron-content coating formulations due to fast radical generation. Measurements were taken again a single time on day 2 and once again at day 4, showing minimal change for the high-iron-content coatings as they had already reached their maximal response. In contrast, the low-iron-content coatings with 30 mg/mL PCDA provide slower radical generation, and thus, could be observed to increase only very slightly within the 4 h period, but at day 2 and day 4 had more pronounced increases in the color change as they had not yet reached their maximal response as they contained less of the catalytic component.

## 4. Discussion

Foremost, we want to highlight that we provide here the first investigation focused on the detection of hydrogen peroxide gas using colorimetric sensor formulations that can be applied by simply spraying onto a surface. To put this in the context of prior work, we have recently demonstrated the use of sprayable diacetylene-containing amphiphile formulations for transforming surfaces into sensors capable of the detection of physical stimuli as well as the detection of chemical target analytes in the liquid phase [23,24]. The work here, in contrast, represents a significant advancement by demonstrating the use of a catalytic component within the spray-coating formulations to enable visual detection of a gas-phase target, specifically hydrogen peroxide. The response that we observed in the distinct spray coating formulations manifests as a clearly observable blue color, which is attributed to the polymerization of the diacetylene monomers comprising the formulation. Directing this response to detect hydrogen peroxide gas is attributed to inclusion of the iron catalyst component at varying amounts of iron(III) chloride, where iron cations have been reported in Fenton-like systems to facilitate the formation of HO_2_• and HO• radical species from hydrogen peroxide, albeit with mechanisms varying widely depending on the system used and specific conditions [27–29]. The generation of free-radical species results in the initiation of the radical polymerization of the diacetylene-containing monomers, wherein the extension of the polymerized diacetylene network facilitates a conjugated pi backbone, exhibiting absorption properties that yield a blue color that is easily observable [30]. In liquid-phase diacetylene-based vesicle systems, several reports of color formation by radical-initiated polymerization have similarly been induced by the presence of radical species formed from hydrogen peroxide decomposition [10,11]. As with those aforementioned works, the examination of the exact mechanism for radical formation and subsequent polymerization is outside of the intended scope of this work; however, redox cycling between iron(III) and iron(II) is known to be facilitated by the presence of hydrogen peroxide and its decomposition products, which are highly system dependent [29,31].

The examination of the factors attributed to tailoring the detection limit of these spray-on sensors provided a clear correlation with the amount of iron(III) chloride incorporated, as well as the amount of the diacetylene-containing amphiphile (PCDA). For a two-hour exposure, a lower detection limit between 3 ppm and 0.2 ppm of hydrogen peroxide vapor could be observed when using higher concentrations of the iron catalyst and PCDA. The response observed when using 10 mM iron(III) chloride was found to saturate within 3.5 to 4 h of exposure to 94 ppm of hydrogen peroxide, suggesting these formulations are most appropriate for cases in which rapid (or acute exposure) testing is desired, as visual color responses were observable within 15 min. In contrast, very low iron(III) chloride content of 0.1 μM of iron(III) chloride within the 30 mg/mL PCDA facilitated long-term assessment of hydrogen peroxide exposure over days rather than hours, thereby extending the utility of this approach to assessing long-term cumulative exposures. Based on a high concentration of 30 mg/mL of PCDA content we can visually observe a blue color within 15 min of exposure to 94 ppm of hydrogen peroxide vapor when using a high iron chloride content of 10 mM. In contrast, that same concentration of PCDA with only 0.1 μM of iron(III) chloride requires between 4 and 22 h of exposure to 94 ppm of hydrogen peroxide vapor to provide a visually observable blue color change due to the slower generation of radical species within the coating. Because the sensor response was found to be dependent on the concentrations of PCDA and iron(III) chloride within the formulations, an important consideration in preparing the coatings is ensuring consistency in the airbrushing of the spray-on sensing material based on factors of distance from substrate, area of substrate to coated, and amount of material to be sprayed.

Because the rate of color change as a function of hydrogen peroxide vapor exposure was dependent on the iron catalyst content of the formulation, we can say definitively that the spray-on sensor response can be tailored for the hydrogen peroxide vapor sensing application for assessing short-term exposure levels (within 15 min) or longitudinal assessment of cumulative exposure (over the course of several days). This is facilitated by the increased blue color intensity observed with increasing cumulative exposure time, which is directly dependent on the amount of iron catalyst present based on our polymerization-based mechanism of detection of the hydrogen peroxide vapor. The spray-on sensor could also demonstrate a unique blue color response depending on the extent of hydrogen peroxide vapor exposure based on the amount of PCDA present, since there is a darker visual response with increasing PCDA content.

## 5. Conclusions

To conclude, this study successfully demonstrated the use of sprayable sensor formulations that enabled the gas-phase detection of hydrogen peroxide vapor. The sensor color response was determined by both the concentration of the diacetylene-containing amphiphile component as well as the amount of iron catalyst. In modulating these formulation parameters, we could enable detection of hydrogen peroxide within minutes or customize the formulation to respond to cumulative exposure over the course of days. Because the color response to different amounts of hydrogen peroxide gas exposure was repeatable and a function of the formulation composition, we could provide the means to detect and quantify the amount of hydrogen peroxide vapor exposure over different durations based on the CIELab colorspace distance. While this work utilized paper as the coating substrate, our previous work has shown that formulations of PCDA without addition of iron(III) chloride could be implemented as color responsive coatings on various substrates including metal, fabric, and plastic [24]. An important consideration in implementing this approach is that diacetylene-containing materials when exposed to UV light are well known to undergo 1,4-addition polymerization to form the conjugated ene-yne alternating backbone that would also result in blue color formation [32]. Thus, the current spray-on sensor formulations for detection of hydrogen peroxide would be limited to being deployed in areas that are not exposed to UV light or placing them in an enclosure which protects against UV exposure that would otherwise interfere with the sensor response by causing a false positive signal. Finally, the prospect of using free-radical-triggered polymerization of diacetylene-containing monomers as a mechanism for detecting target gas molecules is shown here to be effective when the target gas is capable of undergoing catalytic decomposition on the surface resulting in radical formation. Because of this sensing mechanism employing an iron catalyst, this approach could be expanded to other gases, or conversely, from the viewpoint of selectivity, could make the sensor susceptible to blue color changes from compounds other than hydrogen peroxide gas. This would be the case if compounds were present which react with iron to undergo homolytic formation of radical species capable of polymerizing the diacetylene-containing amphiphile components or if reactive radical compounds were otherwise present. In looking forward, future investigations exploring this approach with different catalysts are of interest for detecting a range of different gas targets, and the application of these formulations onto different substrates is exciting and warrants further examination to see if this approach could be used to turn other common substrates into environmental sensors.

## Data Availability

The original contributions presented in the study are included in the article, further inquiries can be directed to the corresponding author.

## References

[1] Nagaraja C; Shashibhushan B; Asif M; Manjunath P Hydrogen peroxide in exhaled breath condensate: A clinical study. Lung India 2012, 29, 123–127. 22628925 10.4103/0970-2113.95303 PMC3354484

[2] Loukides S; Horvath I; Wodehouse T; Cole PJ; Barnes PJ Elevated levels of expired breath hydrogen peroxide in bronchiectasis. Am. J. Respir. Crit. Care Med 1998, 158, 991–994. 9731036 10.1164/ajrccm.158.3.9710031

[3] Brieger K; Schiavone S; Miller FJ Jr.; Krause K-H Reactive oxygen species: From health to disease. Swiss Med. Wkly 2012, 142, w13659. 22903797 10.4414/smw.2012.13659

[4] Blackley BH; Nett RJ; Cox-Ganser JM; Harvey RR; Virji MA Eye and airway symptoms in hospital staff exposed to a product containing hydrogen peroxide, peracetic acid, and acetic acid. Am. J. Ind. Med 2023, 66, 655–669. 37221450 10.1002/ajim.23488 PMC10431326

[5] Giaretta JE; Duan H; Oveissi F; Farajikhah S; Dehghani F; Naficy S Flexible sensors for hydrogen peroxide detection: A critical review. ACS Appl. Mater. Interfaces 2022, 14, 20491–20505. 35486920 10.1021/acsami.1c24727 PMC9104121

[6] Li N; Huang J; Wang Q; Gu Y; Wang P A reaction based one-and two-photon fluorescent probe for selective imaging H_2_O_2_ in living cells and tissues. Sens. Actuators B Chem 2018, 254, 411–416.

[7] Chang MC; Pralle A; Isacoff EY; Chang CJ A selective, cell-permeable optical probe for hydrogen peroxide in living cells. J. Am. Chem. Soc 2004, 126, 15392–15393. 15563161 10.1021/ja0441716 PMC1434465

[8] Guo H; Chen G; Gao M; Wang R; Liu Y; Yu F Imaging of endogenous hydrogen peroxide during the process of cell mitosis and mouse brain development with a near-infrared ratiometric fluorescent probe. Anal. Chem 2018, 91, 1203–1210. 30516972 10.1021/acs.analchem.8b05326

[9] Zheng D-J; Yang Y-S; Zhu H-L Recent progress in the development of small-molecule fluorescent probes for the detection of hydrogen peroxide. TrAC Trends Anal. Chem 2019, 118, 625–651.

[10] Lu S; Jia C; Duan X; Zhang X; Luo F; Han Y; Huang H Polydiacetylene vesicles for hydrogen peroxide detection. Colloids Surf. A Physicochem. Eng. Asp 2014, 443, 488–491.

[11] Jia C; Tang J; Lu S; Han Y; Huang H Enhanced sensitivity for hydrogen peroxide detection: Polydiacetylene vesicles with phenylboronic acid head group. J. Fluoresc 2016, 26, 121–127. 26511954 10.1007/s10895-015-1691-1

[12] Kim M; Shin YJ; Hwang SW; Shin MJ; Shin JS Chromatic detection of glucose using polymerization of diacetylene vesicle. J. Appl. Polym. Sci 2018, 135, 46394.

[13] Maier D; Laubender E; Basavanna A; Schumann S; Güder F; Urban GA; Dincer C Toward continuous monitoring of breath biochemistry: A paper-based wearable sensor for real-time hydrogen peroxide measurement in simulated breath. ACS Sens. 2019, 4, 2945–2951. 31610653 10.1021/acssensors.9b01403 PMC6879172

[14] Oberländer J; Kirchner P; Boyen HG; Schöning MJ Detection of hydrogen peroxide vapor by use of manganese (IV) oxide as catalyst for calorimetric gas sensors. Phys. Status Solidi 2014, 211, 1372–1376.

[15] Lee D-J; Choi S-W; Byun YT Room temperature monitoring of hydrogen peroxide vapor using platinum nanoparticles-decorated single-walled carbon nanotube networks. Sens. Actuators B Chem 2018, 256, 744–750.

[16] Hennemann J; Kohl CD; Reisert S; Kirchner P; Schöning MJ Copper oxide nanofibres for detection of hydrogen peroxide vapour at high concentrations. Phys. Status Solidi 2013, 210, 859–863.

[17] Xu M; Bunes BR; Zang L Paper-based vapor detection of hydrogen peroxide: Colorimetric sensing with tunable interface. ACS Appl. Mater. Interfaces 2011, 3, 642–647. 21355618 10.1021/am1012535

[18] Aroutiounian V; Arakelyan V; Aleksanyan M; Sayunts A; Shahnazaryan G; Kacer P; Picha P; Kovarik J; Pekarek J; Joost B Nanostructured sensors for detection of hydrogen peroxide vapours. Sens. Transducers 2017, 213, 46.

[19] Banga I; Paul A; Muthukumar S; Prasad S HELP (Hydrogen peroxide electrochemical profiling): A novel biosensor for measuring hydrogen peroxide levels expressed in breath for monitoring airway inflammation using electrochemical methods. Biosens. Bioelectron. X 2022, 10, 100139.

[20] Isailović J; Vidović K; Hočevar SB Simple electrochemical sensors for highly sensitive detection of gaseous hydrogen peroxide using polyacrylic-acid-based sensing membrane. Sens. Actuators B Chem 2022, 352, 131053.

[21] Vahidpour F; Alghazali Y; Akca S; Hommes G; Schöning MJ An enzyme-based Interdigitated electrode-type biosensor for detecting low concentrations of H2o2 vapor/aerosol. Chemosensors 2022, 10, 202.

[22] Jannah F; Lee J; Seong H-J; Kim J-M; Kim Y-P A photodynamic color sensor using diacetylene vesicles for the rapid visualization of singlet oxygen. Sens. Actuators B Chem 2023, 380, 133336.

[23] Shiveshwarkar P; Jaworski J Spray-On Colorimetric Sensors for Distinguishing the Presence of Lead Ions. Chemosensors 2023, 11, 327. 38463943 10.3390/chemosensors11060327 PMC10923167

[24] Shiveshwarkar P; Siurano SV; Kadyrova M; Tran N; Jaworski J Investigating the Characteristics and Responses of Diacetylene Based Materials as Spray-On Colorimetric Sensors. Macromol. Res 2022, 30, 1–5.

[25] Manatt SL; Manatt MR On the analyses of mixture vapor pressure data: The hydrogen peroxide/water system and its excess thermodynamic functions. Chem.–A Eur. J 2004, 10, 6540–6557. 10.1002/chem.200400104 15551324

[26] Scatchard G; Kavanagh GM; Ticknor LB Vapor-Liquid Equilibrium. VIII. Hydrogen Peroxide—Water Mixtures1. J. Am. Chem. Soc 1952, 74, 3715–3720.

[27] Zhao L; Lin Z-R; Ma X.-h.; Dong Y-H Catalytic activity of different iron oxides: Insight from pollutant degradation and hydroxyl radical formation in heterogeneous Fenton-like systems. Chem. Eng. J 2018, 352, 343–351.

[28] Burns JM; Craig PS; Shaw TJ; Ferry JL Multivariate examination of Fe (II)/Fe (III) cycling and consequent hydroxyl radical generation. Environ. Sci. Technol 2010, 44, 7226–7231. 20469853 10.1021/es903519m

[29] Meyerstein D Re-examining Fenton and Fenton-like reactions. Nat. Rev. Chem 2021, 5, 595–597. 37118415 10.1038/s41570-021-00310-4

[30] Yoon B; Jaworski J; Kim J-M Size-dependent intercalation of alkylamines within polydiacetylene supramolecules. Supramol. Chem 2013, 25, 54–59.

[31] Koppenol WH The Haber-Weiss cycle–70 years later. Redox Rep 2001, 6, 229–234. 11642713 10.1179/135100001101536373

[32] Qian X; Städler B Polydiacetylene-Based Biosensors for the Detection of Viruses and Related Biomolecules. Adv. Funct. Mater 2020, 30, 2004605.

